# *Eremonidiopsis aggregata*, gen. n., sp. n. from Cuba, the third West Indian Dioptinae (Lepidoptera, Notodontidae)

**DOI:** 10.3897/zookeys.333.5483

**Published:** 2013-09-20

**Authors:** Rayner Núñez Aguila

**Affiliations:** 1 División de Colecciones Zoológicas y Sistemática, Instituto de Ecología y Sistemática, Carretera de Varona km 3. 5, Capdevila, Boyeros, La Habana, Cuba. CP 11900. Habana 19

**Keywords:** Taxonomy, Lepidoptera, Noctuoidea, Notodontidae, Dioptinae, Dioptini, West Indies, Cuba, aggregation, conservation

## Abstract

A new genus and species of Dioptinae (Lepidoptera, Noctuoidea, Notodontidae) is described from Cuba, this being the third taxon of the subfamily known from the West Indies. *Eremonidiopsis aggregata*, gen. n., sp. n., appears to be closely related to *Eremonidia mirifica* Rawlins & Miller from Hispaniola among members of the tribe Dioptini. *Eremonidiopsis aggregata* is known from two localities in the middle and western portions of the northeastern Cuban mountain range, Nipe–Sagua–Baracoa. The species inhabits low elevations (300–400 m) covered by lowland rainforest and sclerophyll rainforest. The six known specimens, all males, were part of small swarms flying near the top of an unidentified tree during the day at both collecting sites. These localities are included within protected areas, the “Pico Cristal” National Park in the West and the “Alexander von Humbolt” National Park in the East.

## Introduction

The Dioptinae (Notodontidae) includes 466 species, all but one Neotropical (Miller 2009; Miller and Thiaucourt 2011). Until the present work, this subfamily was represented in the West Indies by two species placed in two endemic genera, *Eremonidia* Rawlins & Millerand and *Caribojosia* Rawlins & Miller, from the island of Hispaniola (Rawlins and Miller 2008). In the same article, the authors mentioned the possible presence of other members of this subfamily above 1500 m in the Blue Mountains and Sierra Maestra mountain ranges, of Jamaica and Cuba respectively.

In the present paper, a new genus of Dioptinae is described from Cuba. As predicted by Rawlins and Miller (2008), this taxon was found in eastern Cuba but in the northeastern mountain range, Nipe–Sagua–Baracoa, instead of the southern Sierra Maestra. The systematic position of the new taxon is discussed and observations on its habitat and behavior are also described.

## Materials and methods

Characters for genus and species descriptions are the same as those used by Rawlins and Miller (2008) and Miller (2009).

Genitalia were dissected by maceration in 10% potassium hydroxide, later neutralized in 30% alcohol with two drops of glacial acetic acid, and finally stored in glycerin in microvials. Wings were cleared in sodium hypochlorite bleach, stained with Eosin-Y, and slide-mounted in Euparal.

Type material is deposited at the entomological collection of the Institute of Ecology and Systematics (CZACC), Havana, Cuba. Updated information on species richness and endemism on other Lepidoptera groups at the Nipe–Sagua–Baracoa mountain range was obtained from the CZACC collection and the literature is cited in each case.

### Other abbreviations used

AHNP “Alexander von Humboldt” National Park

CuA cubito–anal vein

FW forewing

HW hindwing

M medial vein

NSB Nipe–Sagua–Baracoa

R radial vein

Rs radial sector

## Results

### Systematics

#### Eremonidiopsis gen. n.

http://zoobank.org/AE08ED3D-1B56-4580-81B6-514C840F310F

http://species-id.net/wiki/Eremonidiopsis

##### Type species.

*Eremonidiopsis aggregata* Núñez, new species, by monotypy.

##### Diagnosis.

*Eremonidiopsis* can be recognized by a combination of the following characters: antennae bipectinate; FW veins Rs2–Rs4 branch in the pattern Rs2+[Rs3+Rs4]; FW discal cell very long, about 65% of FW length; male without stridulatory organ, FW veins M1 and M2 not swollen at their bases; veins M3 and CuA1 separate in the FW and stalked in the HW.

*Eremonidiopsis* appears to be a close relative of *Eremonidia* from Hispaniola, one the two other known West Indian Dioptinae genera (Rawlins and Miller 2008; Miller 2009). The stalk of Rs1 with Rs2–Rs4 branch is long in *Eremonidia* but in *Eremonidiopsis* arises just after the origin from the discal cell or is even connate. They also differ by the color of the proboscis, golden brown in *Eremonidia* and blackish brown in *Eremonidiopsis*, and in the size head respect to insect size. Both taxa show a similar size, with a FW length of 12.7 mm in *Eremonidia mirifica* and 12.2 mm in *Eremonidiopsis aggregata*; however, head width across the eyes is 1.41 mm in the latter whereas in the Hispaniolan genus the measure is 1.77 mm or 25% larger. The tympanum also exhibits differences. The membrane is enclosed, deep, and oriented horizontally in *Eremonidia* (Rawlins and Miller 2008; Miller 2009) whereas in *Eremonidiopsis* it is shallow, not enclosed, and oriented vertically. Although their male genitalia show similarities when compared to other Dioptinae, they exhibit differences in shape of the valvae, aedeagus, and anal tube, as well as in possession by *Eremonidiopsis* of dorsolateral keels on the uncus. Finally, the shape of the male eight sternum differs as well the male seventh sternum which is modified only in *Eremonidiopsis aggregata*.

Compared to other Dioptinae, *Eremonidiopsis* is distinctive by having FW veins M3 and CuA1 separate, whereas in most Dioptinae these veins are stalked (Miller 2009). The radial system branching pattern also differs from the typical Dioptinae one, [Rs2+Rs3]+Rs4 (Miller 2009). *Eremonidiopsis* exhibits a color pattern similar to some species of *Scotura* Walker, 1854; however, the latter possesses ciliate male antennae, a shorter FW discal cell, and a stridulatory organ, among others differences.

The phylogenetic position of the new genus will be better understood when females and larvae are available. Although some characters suggest a relation with *Eremonidia*, the lack of FW stridulatory organ and different tympanum of *Eremonidiopsis* imply that may be is closer to some other clade within the Dioptinae.

##### Description.

**Male.** *Head*. Labial palpus short and thin, curved strongly upward to just above clypeus, held close to front; first segment moderate in length, curved upward; second segment slightly shorter than first segment; third segment short, conical, pointed at apex; labial palpus ratio 1/0.85/0.20; proboscis blackish brown; scales of front short, appressed and directed dorso-medially, a pair of small tufts between antennal bases and eyes; eyes moderately large, bulging; vertex covered with semi-erect scales; antennae bipectinate, each flagellomere bearing a basal pair of ciliate rami; rami longer at middle segments, about 3.5 times length of supporting flagellomere; flagellomeres 35–37. *Thorax*. Epiphysis long, equal in length to tibia; tibial spurs moderate in length, apical pair half as long as basal pair on metathoracic tibia; tegulae covered with long scales, outer margins fringed with hairlike scales; tympanum large, rounded, cavity shallow; tympanal membrane facing posteriorly. Forewing elongate, apical angle slightly acute; vein R1 arising from discal cell; Rs1 connate or stalked just after origin with Rs2–Rs4; veins Rs2–Rs4 in pattern Rs2+[Rs3+Rs4]; M1 separate from radial sector; stridulatory organ absent; discal cell about 65% length of wing; M3 widely separate from CuA1. Hindwings broad, outer margin expanded; apical angle rounded; vein M3 short stalked with CuA1; discal cell 60% length of wing. *Abdomen*. Short, gradually tapered, with a small, inconspicuous distal tuft of moderately long scales. Eighth tergum large, more than twice length of seventh tergum, slightly narrower posteriorly; eighth sternum relatively short, narrower than seventh sternum, anterior margin bearing a slightly elongate, sac-like apodeme. Seventh sternum with lateral margins curved, gradually tapering toward anterior margin, which is sclerotized and bears a short anteriorly directed mesal process.

*Genitalia*. Socii/uncus complex moderate in size, heavily sclerotized, narrowly joined to arms of tegumen; arms of tegumen relatively wide, much taller than vinculum; arms of vinculum short and wide; valve narrow, Barth’s Organ absent; costal and ventral margin of valve sclerotized, each folded toward inner surface with a sclerotized low flange; inner surface of valve concave, with scattered coarse setae; arms of transtilla sclerotized and narrow, oriented horizontally, with a pair of short acute processes anteriorly and a wide sclerotized ventral plate; juxta large, dorsal margin with a shallow mesal excavation; aedeagus large, thin and cylindrical, base greatly expanded; apex of phallus curved downward, spoon shaped; opercular sclerite absent; vesica moderately long, much shorter than aedeagus, bent slightly upward; vesica bearing a large mass of deciduous caltrop cornuti along ventral surface, these varying in spine length.

**Female**. Unknown.

##### Etymology.

The generic name *Eremonidiopsis* is derived from the name of its Hispaniolan relative *Eremonidia*. The suffix –*opsis* refers to the resemblance of the Cuban genus to the Hispaniolan one.

##### Distribution.

The six known specimens were captured at two localities in different sections at the western half of the NSB mountain range in northeastern Cuba.

##### Immature stages.

Unknown.

##### Remarks.

This taxon and *Eremonidia* are evidently close relatives. They share several characteristics including the short labial palpi, similar wing venation (FW radial system pattern Rs2+[Rs3+Rs4], a long FW discal cell, and veins M3 and CuA1 separate in the forewing but stalked in the hindwing), as well as several features of the male genitalia, which are highly divergent from the remaining Dioptini (Miller 2009). These similarities suggest placement of *Eremonidiopsis* close to *Eremonidia* in the basal clade of the Dioptini (Miller 2009). Available evidence shows few features linking *Eremonidiopsis* to the remaining members of this clade: *Scotura*, *Cleptophasia* Prout, 1918, *Oricia* Walker, 1854, and *Erbessa* Walker, 1854 (Miller 2009). The latter shares the possession of a shallow tympanum whereas *Cleptophasia* possesses a long FW discal cell. As in *Eremonidia*, the possession of large, deciduous caltrop cornuti on the vesica of males indicates a plesiomorphic phylogenetic position (Rawlins and Miller 2008).

#### Eremonidiopsis aggregata sp. n.

http://zoobank.org/0EA20D76-403F-4526-BB2A-8CB2AF0D9AC2

http://species-id.net/wiki/Eremonidiopsis_aggregata

Figs 1 –7

##### Type material.

Holotype: ♂, Cuba, Holguí, Moa, vicinity of Morones mountain stream (20°26'22"N, 74°49'14"W), 300 m, 22/V/2007, R. Núñez. Paratypes: 5 ♂. Same data as holotype (4 ♂); Holguín, Mayarí, vicinity of La Zoilita (20°37'42"N, 75°29'08"W), 400 m, 6/IV/2012, R. Núñez (1 ♂).

##### Diagnosis.

The uniform dark brown wing pattern of *Eremonidiopsis aggregata* is present only in *Scotura nigricaput* Dogninand *Scotura flavicapilla* (Hübner) among all Dioptinae. *Eremonidiopsis aggregata* can be easily separated from the first by its yellowish-orange collar and from the second by lacking yellowish color at other areas of the head such as the front and the vertex. In addition, many other features allow separation of *Eremonidiopsis* from *Scotura* including the possession of bipectinate antennae, a longer discal cell, absence of the FW stridulatory organ, and absence of the Barth’s Organ in the male genitalia among other features.

##### Description.

**Figure 1.**
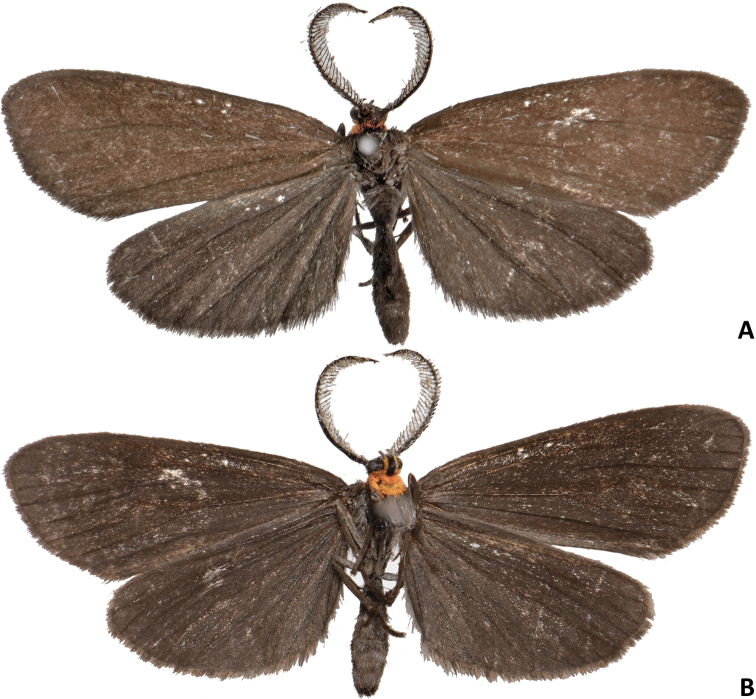
Habitus of *Eremonidiopsis aggregata*. **A** Male holotype, dorsal view **B** Male holotype, ventral view.

**Figure 2.**
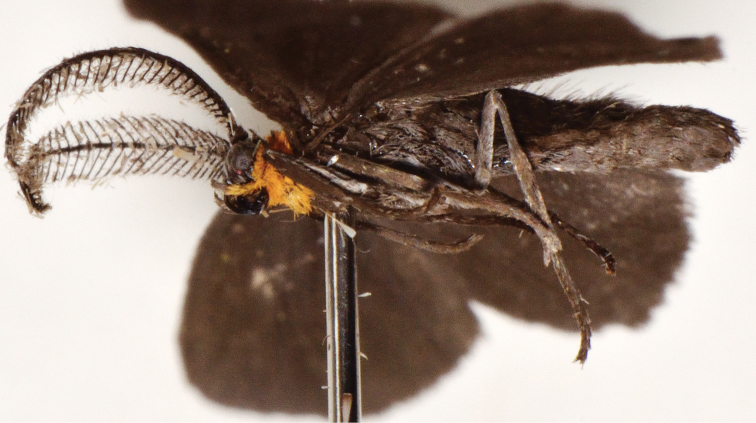
Head, thorax, and abdomen of *Eremonidiopsis aggregata*, lateral view.

**Figure 3.**
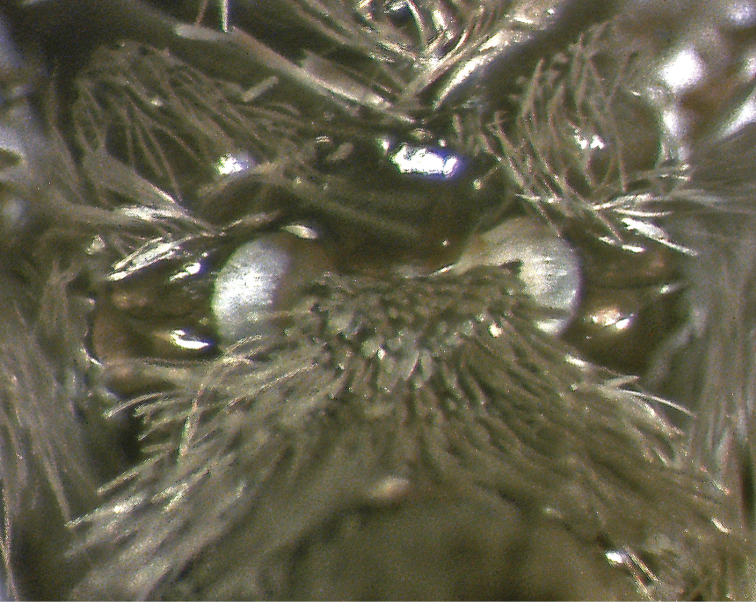
Tympanum of *Eremonidiopsis aggregata*, posterior view of metathorax.

**Figure 4.**
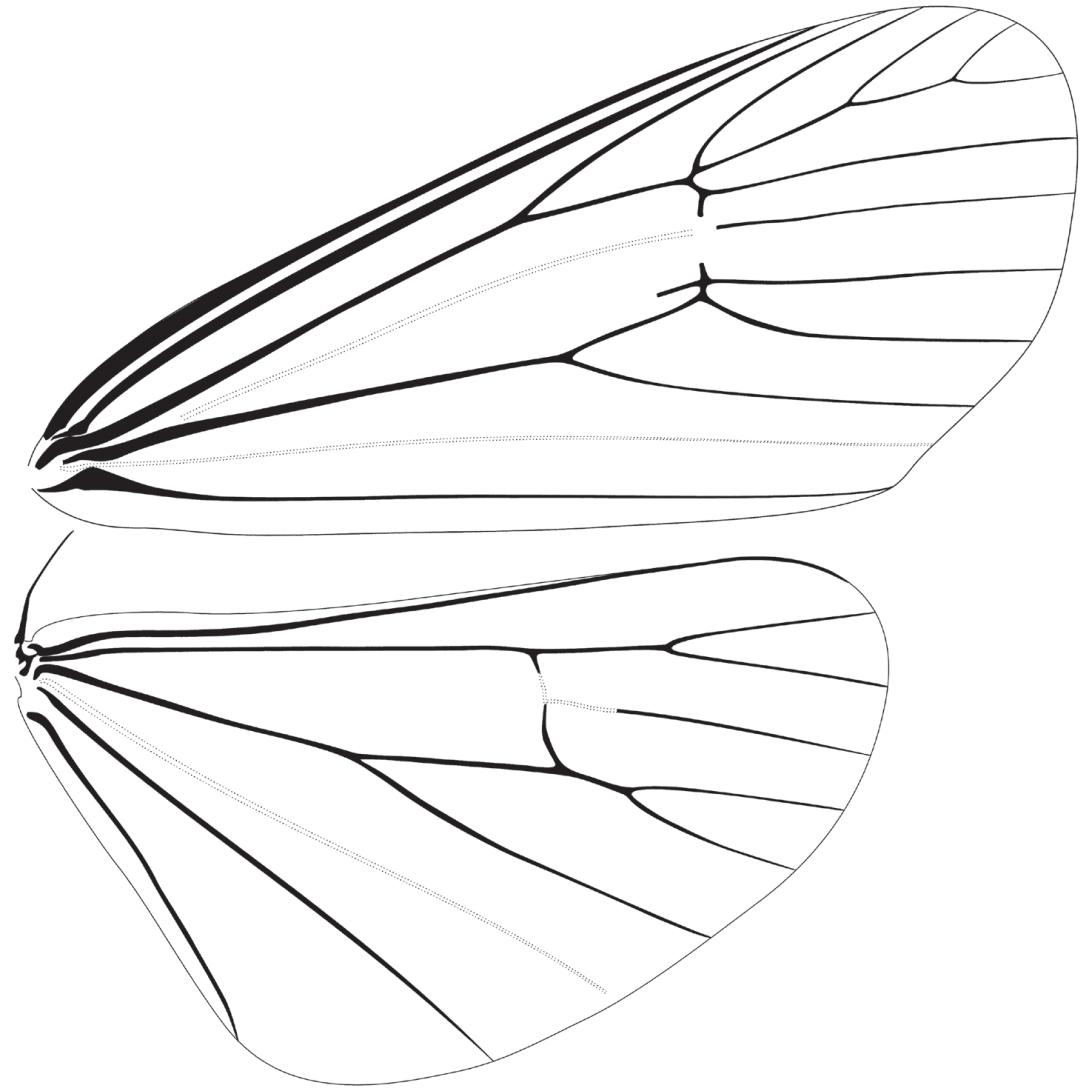
Wing venation of *Eremonidiopsis aggregata*.

**Figure 5.**
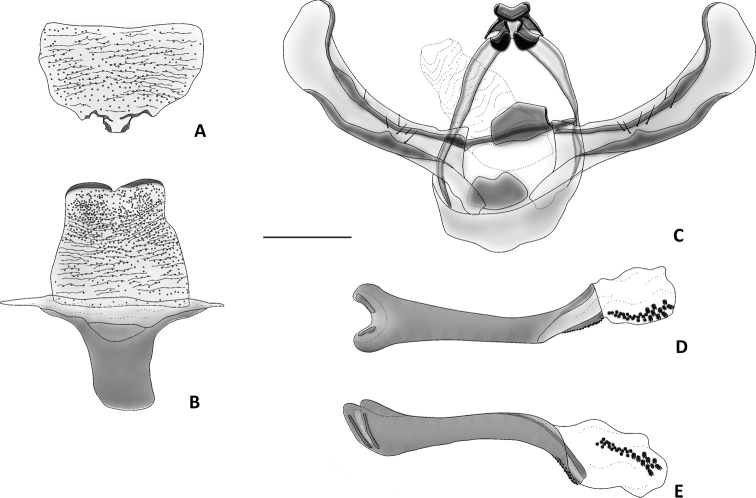
Seventh and eighth abdominal sterna and male genitalia of *Eremonidiopsis aggregata*. Scale bar 0.5 mm. **A** Seventh sternum, dorsal view **B** Eighth sternum, dorsal view **C** Genitalia, ventral view **D** Aedeagus, dorsal view **E** Aedeagus, lateral view.

**Male** (Figs 1–5). *Head*. First segment of labial palpus covered with short, yellowish-orange scales; third and second segments of labial palpi brownish gray, second segment with scattered yellowish-orange scales on inner side; remaining parts of head covered with appressed, glossy brownish-gray scales. Eyes moderately large, measurements (N=6), mean ± S.D. (range); width of head across eyes: 1.41 ± 0.02 mm (1.38–1.43 mm); height of eye: 0.57 ± 0.02 mm (0.53–0.58 mm); ocular index (height of eye / width of head): 0.40 ± 0.01 mm (0.38–0.41 mm). *Thorax*. Propleuron and prosternal region yellowish orange between base of proboscis and base of brownish-gray procoxae (Figs 1, 2); dorsum brownish gray; venter, including legs, pale brownish gray except inner side of femora grayish white; tympanum contrastingly dirty white (Fig. 3). Forewing (Figs 1, 4) with dorsal surface glossy, uniformly brown; ventral surface uniformly brownish gray; measurements (N=6), mean ± S.D. (range); length: 12.2 ± 0.24 mm (12.0–12.7 mm); width: 5.5 ± 0.22 mm (5.2–5.8 mm); length / width ratio: 2.2 ± 0.05 (2.2–2.3). Hindwing (Figs 1, 4) with dorsal surface uniformly dark brownish gray; ventral surface uniformly brownish gray (Fig. 1); measurements (N=6), mean ± S.D. (range); length: 9.5 ± 0.35 mm (9.1–10.1 mm); width: 5.5 ± 0.11 mm (5.2–5.5 mm); length / width ratio: 1.8 ± 0.04 (1.75–1.84). *Abdomen*. Scales of dorsum glossy, brownish gray; venter white brownish gray but paler (Figs 1, 2). Mesal process on anterior margin of seventh sternum short and blunt, flanked by indentations (Fig. 5B). Eighth tergum elongate, slightly longer than corresponding sternum excluding apodeme; anterior margin one third broader than posterior one, slightly excavated at middle; lateral margins simple; posterior margin slightly convex with a short hood-shaped fold. Eighth sternum with lateral margins simple; posterior margin sclerotized except at shallow mesal excavation; anterior margin one third broader than posterior one (Fig. 5A); anterior apodeme saclike, about two thirds as long as sternum, gradually tapering toward rounded anterior end, lateral margins simple. *Genitalia*. Uncus short, wide, curved gradually downward, dorsum convex, apex acute, with a pair of triangular dorsolateral keels; socii short, wide at bases, curved strongly upward, apices cup shaped (Fig. 5C); dorsal portion of tegumen gently tapered, ventral portion slightly widened at junction with vinculum; saccus broad, quadrate, ventral margin transverse, dorsal margin wide, slightly convex, barely covering valve bases; inner surface of valve mostly membranous; dorsal margin of costa slightly convex with a low sclerotized flange on inner surface extending to apex, with a blunt expansion in apical third (Fig. 5C); ventral margin of valve mostly straight, folded toward inner surface to form a low sclerotized flange in distal third, flange with a blunt expansion at middle and a more acute one near apical third; apical portion of valve broadly expanded and rounded (Fig. 5C); anal tube short and broad, extending below apex of valvae; apex of aedeagus dentate along right lateral margin, teeth heavily sclerotized (Fig. 5D); caltrop cornuti bearing three or four straight upwardly oriented spines, longest spines up to 5 × length of shortest ones.

**Female**. Unknown.

##### Etymology.

The species–group name is derived from the Latin *gregis* (flock, group) and the suffix *atus* (having the nature of), in reference to the aggregation of individuals observed during both collecting events.

##### Distribution

**Figure 6.**
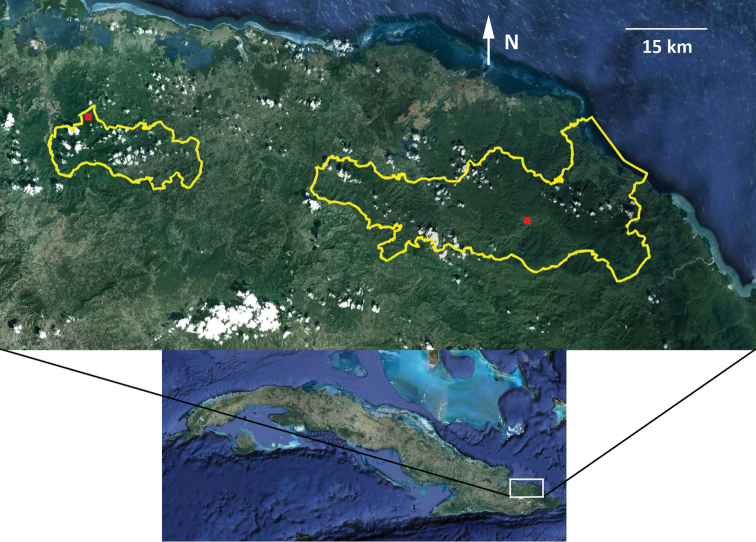
Distribution of *Eremonidiopsis aggregata* at the middle and western portions of the Nipe–Sagua–Baracoa range, northeastern Cuba. Right red square indicating the vicinity of the Morones mountains stream, left red square indicating the vicinity of La Zoilita. Yellow polygons showing area occupied by National Parks, “Alexander von Humboldt” at the right, and “Pico Cristal” at the left.

(Fig. 6). Known from only two localities of the NSB mountain range, both in Holguín province, northeastern Cuba. The locality at the center of the NSB is in the vicinity of Morones mountain stream (20°26'22"N, 74°49'14"W; 300 m) near the Jaguaní river east of La Melba village on the southeastern slope of the El Toldo plateau. The westernmost locality is the vicinity of La Zoilita (20°37'42"N, 75°29'08"W; 400 m), on the northern slope of Sierra de Cristal.

##### Habitat

**Figure 7.**
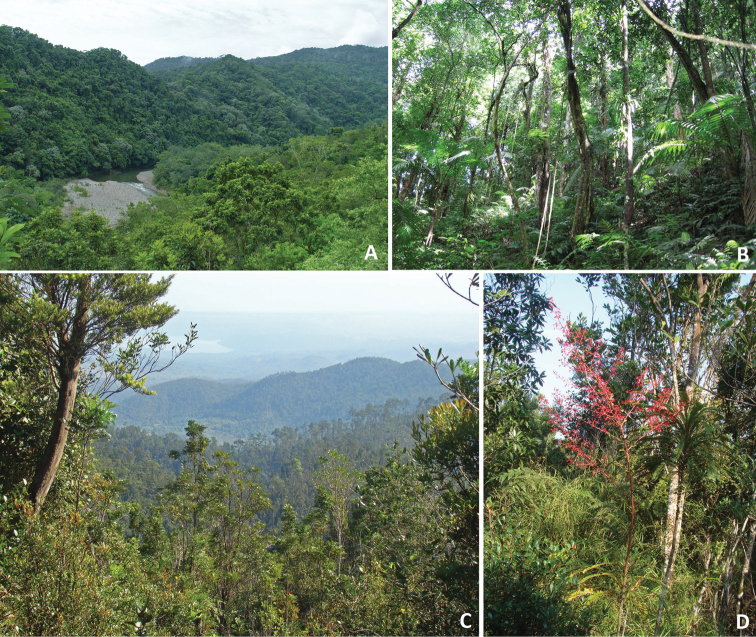
Habitats of *Eremonidiopsis aggregata* in Nipe–Sagua–Baracoa range, northeastern Cuba. **A** Lowland rainforest at the riversides of the Jaguaní River at La Melba, near the type locality **B** Understory of the lowland rainforest in the vicinity of Morones mountain stream **C** Northern slopes of Sierra Cristal, near the type locality **D** Sclerophyll rainforest in the vicinity of La Zoilita.

(Fig. 7). The two localities where *Eremonidiopsis aggregata* has been collected are very different regarding both vegetation and climate. Vegetation around La Melba is represented by lowland rainforest, Cuba’s most exuberant rainforest type (Figs 7A, B) (Reyes and Acosta 2005). The vegetation has a distinctly mesophyllic aspect due to the predominance of *Carapa guianensis* Aubl. (Meliaceae). Generally, there are two arboreal layers; the upper layer frequently reaches 30 to 35 m; when it only reaches 20 to 25 m, it has emergent individuals reaching 35 m (Reyes and Acosta 2005). Arboreal canopy coverage is 100%. A more detailed description of this rainforest is given by Reyes and Acosta (2005). Rainfall is 3400 mm per year; this is the rainiest region of Cuba with up to 240 rainy days per year (Montenegro 1991; Zabala and Villaverde 2005). October to January and May are the wettest periods, averaging 300–500 mm of rainfall per month; February and March are the least rainy months with about 200 mm (Montenegro 1991). Cuba’s highest relative humidity rates occur there; yearly values vary between 90 and 95%. The most humid month is October and the least humid month is July. Temperatures are high, between 22 and 26°C, which along with frequent and long calm periods produce a sensation of suffocating heat (Montenegro 1991).

The substratum rocks are metamorphic. Soils are poor, acidic, and humid (“Ferralítico Rojo Lixiviado” and “Ferralítico Amarillento Lixiviado”), over a ferralitic, meteorized (weathered) crust (Zabala and Villaverde 2005).

The vicinity of La Zoilita is covered by sclerophyll rainforest [name modified from Borhidi (1991) following Reyes and Cantillo (2005)] (Figs 7C, D). Leaves are very sclerophyllous, mostly microphyll and notophyll. The arboreal layer is open and irregular in height and generally fluctuates between 15 and 20 m. Constant and abundant species include *Calophyllum utile* Bisse (Clusiaceae), *Guapira rufescens* (Heimerl) Lundell (Nyctaginaceae), and *Tabebuia dubia* (C. Wright ex Sauvalle) Britton ex Seibert (Bignoniaceae). The lower stratum is 5–12 m high and more closed. Details concerning the structure and species composition of this habitat can be found in Borhidi (1991) and Reyes and Cantillo (2005).

Rainfall is 1600 mm per year. Average annual relative humidity is 91% at 7:00 am, and 67% at 1:00 pm. The average yearly temperature is 21.6°C, with the highest average value being 23.8°C during July, and the minimum being 19.0°C in February.

The substratum rocks are ophiolithic. The soils are “Ferríticos Rojos Oscuros,” very poor and acidic, and shallow to very deep; sometimes with bare rock exposed.

##### Behavioral observations.

All individuals of *Eremonidiopsis aggregata* were observed in flight during the early afternoon. At both localities the species was found in small, agitated swarms of 10 to 15 individuals. Flight was moderately strong and erratic, and on both occasions the moths were seen flying 3 to 4 meters above the ground around the top of an unidentified tree. Specimens were captured when they occasionally descended near the ground. No females where captured.

Roughly 60 hours of light trapping were spent at La Zoilita using a 250 watt mercury vapor bulb in February of 2010, but no *Eremonidiopsis aggregata* specimens were attracted. However, individuals were collected there during the day in April and May in the vicinity of Morones mountain stream.

## Discussion

The NSB range contains the largest and best-preserved mountain ecosystem remnants in Cuba and possibly in all of the Caribbean islands (Fong et al. 2005). With a maximum altitude of 1231 m (Pico Cristal), the NSB, which occupies an area of 9350 km^2^, is the largest Cuban mountain range (C.N.N.G. 2000). The diversity of vegetation types (rainforests, pine forests, evergreen forests, and charrascals) cover large areas in a complex mosaic resulting from a multitude of soil types, as well as differences in humidity, sun exposure, and altitude (Fong et al. 2005).

NSB harbors the highest values of species richness and endemism in the Cuban flora and fauna for many groups including liverworts, mosses, vascular plants, spiders, hymenopterans, amphibians and reptiles (Sánchez-Ruiz 1999; Mustelier 2001; Portuondo and Fernández 2004; Fong et al. 2005). Regarding Lepidoptera, Alayón and Solana (1987) listed 113 butterfly species from the “Cuchillas del Toa” Biosphere Reserve. Today, with the addition of another 39 records (Smith and Hernández 1992; Hernández et al. 1998; Reyes and Núñez 2006; Núñez 2007, 2010; Núñez et al. 2012, 2013), there are 152 species known from NSB. That number represents 78% of all Cuban butterflies (Núñez and Barro 2012; Núñez et al. 2013). Endemism is also very high; 28 of the 33 butterflies exclusive to Cuba are present in that mountain range. The picture is similar for two of the best-known moth families, Notodontidae and Sphingidae. Twenty notodontid species have been recorded there, 71% of the Cuban total, and 8 of the 9 endemics (Torre and Alayo 1959; Núñez and Barro 2012; this work). The Sphingidae is represented by 38 species, 63% of the Cuban fauna, and 9 of the 14 endemic species (Zayas and Alayo 1956; Reyes et al. 2003; Haxaire and Melichar 2012; Núñez and Barro 2012).

The presence of *Eremonidiopsis* at NSB is not completely unexpected given the high Lepidoptera diversity mentioned above, as well the presence of Dioptinae on the neighboring Hispaniola (Rawlins and Miller 2008; Miller 2009). It has been well established that eastern Cuba and north–central Hispaniola were joined or in close proximity during the Paleocene–Eocene (Pindell and Barrett 1990; Draper and Barros 1994; Iturralde-Vinent and MacPhee 1999). Perhaps the Antillean Dioptinae ancestors evolved when the land masses separated from one another. This hypothesis could explain the close relationship between *Eremonidiopsis* and *Eremonidia*. A similar theory has been suggested for butterflies in the endemic West Indian genus *Calisto*, Nymphalidae: Satyrinae (Miller and Miller 2001; Núñez et al. 2012).

There is little information on the biology of *Eremonidiopsis aggregata*. Diurnal activity is common throughout the Dioptinae, although there are also strictly nocturnal species as well as others that fly both at night and during the day (Miller 2009). In the case of *Eremonidiopsis aggregata* present data suggest that the species is diurnal; light collecting was performed at one of the type localities and in similar locations at NSB without success in attracting specimens.

Observed clustering during flight seems to be one of the few records of this behavior in the Dioptinae. The small size of *Eremonidiopsis aggregata* and the flight’s height precluded more detailed observations. Possible explanations include that individuals were feeding on tree flowers or that they were engaging in some kind of courtship event, though neither have been reported for the Dioptinae.

Regarding potential hostplants, many of the genera used by the Dioptinae listed in Miller (2009) are present in Cuba. Among the plants used by members of the basal clade of Dioptini, where *Eremonidiopsis* is potentially placed, species of *Hybanthus* Jacq. (Violaceace), *Acalypha* L. (Euphorbiaceae), *Acacia* Mill. (Fabaceae), *Meliosma* (Meliosmaceae), *Genipa* L. and *Randia* L. (Rubiaceae), *Eugenia* L. (Myrtaceae), and *Miconia* Ruiz & Pav., *Conostegia* D.Don, and *Henriettea* DC. (Melastomataceae) are present in Cuba (Acevedo-Rodríguez and Strong 2012). Of these, *Eugenia*, *Miconia*, and *Henriettea* are well represented in the NSB range with 31 species of the three plant genera, including 21 endemics, recently recorded from AHNP (Martínez et al. 2005).

Concerning conservation, both collection localities are included within protected areas. The Morones mountain stream runs through the heart of the AHNP. This park, with 706.8 km^2^, is one of Cuba’s largest and most important protected areas (Category II, World Conservation Union, IUCN) in terms of biodiversity. The AHNP constitutes the core of the “Cuchillas del Toa” Biosphere Reserve. La Zoilita, with 185 km^2^, is within the “Pico Cristal” National Park, and its eastern limit is located 20 km from the western border of AHNP.

*Eremonidiopsis aggregata* probably occurs across NSB at any location where habitats in good condition persist. Collections of Lepidoptera from the NSB range with vouchers at CZACC date from the first decade of the twentieth century. However, localities are mostly grouped around the towns of Baracoa and Moa. *Eremonidiopsis aggregata* remained undiscovered and absent from collections probably due to a combination of its small size and dark, non–attractive coloration, as well as perhaps to its rarity.

## Supplementary Material

XML Treatment for Eremonidiopsis

XML Treatment for Eremonidiopsis aggregata
